# Short Spatiotemporal Fire History Explains the Occurrence of Beetles Favoured by Fire

**DOI:** 10.3390/insects15100775

**Published:** 2024-10-07

**Authors:** Per Milberg, Karl-Olof Bergman, Nicklas Jansson, Henrik Norman, Fia Sundin, Lars Westerberg, Victor Johansson

**Affiliations:** IFM Biology, Conservation Ecology Group, Linköping University, SE-581 83 Linköping, Sweden

**Keywords:** Coleoptera, forest fire, pyrophilic, conservation burns

## Abstract

**Simple Summary:**

How far in space and time should conservation burns be conducted to provide most benefit to beetles favoured by forest fires? We systematically sampled forest reserves with different fire history and found that most pyrophilic beetles were found when fires in the vicinity of the reserves were close and quite recent.

**Abstract:**

The number and area of forest fires in northern Europe have been dramatically reduced during the past century, and several fire-favoured species are now threatened. To promote the recovery of these species, prescribed burning is often used as a conservation measure, and to optimise the use of these conservation burns, knowledge is needed on suitable fire frequency, size and placement in the landscape. The aim of this study was to analyse the effect of recent fire history (12 yrs) on beetles sampled using smoke attraction traps at 21 forest sites in a 10,000 km^2^ region. We analysed the odds of finding a fire-favoured beetle species or individual among the beetles in each trap using a new spatiotemporal connectivity measure and compared the results to non-fire-favoured and saproxylic species. For fire-favoured beetles, both the number of species and individuals significantly increased with connectivity to previous fires, while the other two groups did not. The spatiotemporal connectivity that best explained the patterns suggests that fire-favoured beetles mainly respond to fires within a 2 km range up to 2–3 years after the fire. Hence, to preserve fire-favoured insects, prescribed fires must be close in space and time to other fires—whether prescribed or natural.

## 1. Introduction

Fires were previously a common feature in the boreal forests of Scandinavia [1,2] and have exerted a strong selection force on many forest organisms [3,4] including plants, fungi, beetles, flies and other arthropods [5,6,7,8,9,10,11,12,13]. Species with clear morphological adaptation to fires are considered “pyrophilic”, while the term is sometimes more loosely applied to species that are rare in the absence of fire (see [4] for a review). Together, these two groups could be called “fire-favoured”. In the current paper, we use “pyrophilic” in its broad sense, meaning fire-favoured. In boreal forests, pyrophilia might have once been relatively prevalent compared to today, and several studies have shown immediate and massive colonisation of recent burns by insects [14,15,16,17]. Some of these species are attracted to the amount of deadwood as well as stressed, dying, or recently dead trees, typical in recently burned habitats [18]. The numbers and area of forest fires in northern Europe have dramatically decreased in the past century [2,19,20,21], and this has been linked to the decline seen in some species [22,23,24,25]. To counteract this loss and restore the lost fire regimes, people are now using prescribed burning.

Conservation burns, i.e., prescribed burns conducted with the purpose of promoting biodiversity rather than reducing fuel loads, can be an efficient conservation measure in intensively managed boreal forest landscapes [26], as it can quickly transform an intensively managed forest stand to a suitable habitat for rare and threatened species. Prescribed forest fires are currently used as a conservation tool in protected forests in Fennoscandia [7,27], with the forestry certification systems FSC (Forest Stewardship Council) and PEFC (Programme for the Endorsement of Forest Certification) creating momentum by demanding that owners of forests in Sweden burn 5% of the regeneration area on dry and mesic ground if their property is larger than 5000 ha [27]. Conservation burns have been shown to have profound positive effects on the general species assemblage and both fire-dependent and other less fire-specialised forest species benefit from fires [16,17,28]. However, the ecological benefit of conservation burns for these species depends on the availability of habitat patches that harbour potential colonisers for the newly burned areas. This availability is partly determined by the forest fire history and the habitat quality of the surrounding landscape [26]. For example, fire history seemed to promote some pyrophilic Diptera and Coleoptera [29]. To optimise the use of conservation burns, knowledge is needed on suitable fire frequency, size of fires and placement in the landscape.

The aim of the present study was to analyse the occurrence of beetles in relation to short-term forest fire history (12 yrs preceding sampling). As burnt areas quickly lose much of their unique features [30], the connectivity of burnt habitats involves a temporal dimension in addition to the spatial one. Therefore, we developed a spatiotemporal connectivity measure that was then applied to pyrophilic beetle species. As a control, we also analysed non-pyrophilic species, expected to occur irrespective of forest fires, as well as obligate saproxylic ones, expected to depend on forest cover. Sampling was done in protected forests using smoke attraction trapping, a method for potentially catching pyrophilic insects without an actual forest fire [29]. A previous study involved preliminary analyses that indicated a positive relationship between the area of forest previously burnt near the sampling points and occurrence of pyrophilic beetles [29]. Hence, we evaluated the hypothesis that there is a relationship between spatiotemporal connectivity to fires and finding pyrophilic beetles.

## 2. Methods

### 2.1. Study Area

**Figure 1:**
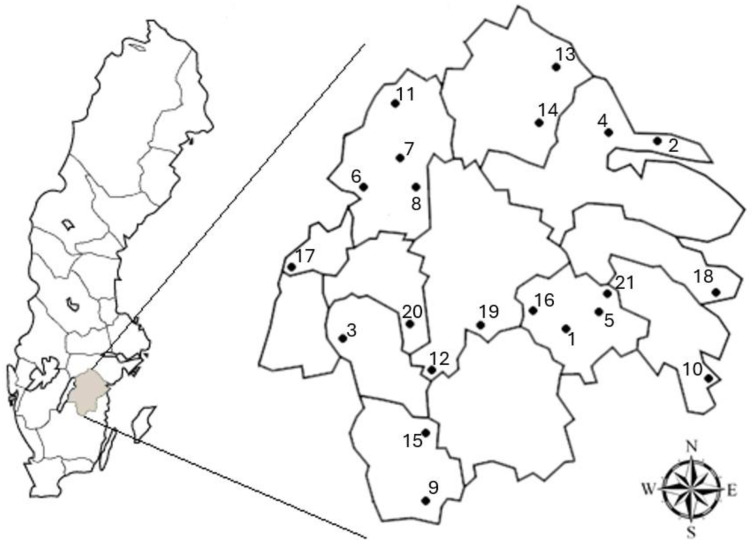
Location of the 21 study sites where smoke attraction trapping was used to catch insects in forests in Östergötland county, Sweden. Borders show the 13 municipalities within the county; n.b. that fire statistics from 11 municipalities in adjoining counties were included.

We used smoke attraction trapping in field work conducted between 14 June and 31 July 2011. This study involved 21 forest sites in Östergötland County, south-east Sweden (Figure 1). All sites were in protected forests. The sites were selected based partly on a previous analysis of the forest fire history in the county [31] to include sites with a wide range of forest fire densities in the surrounding landscape. To reduce spatial dependence, sites were located more than 7 km away from each other. This cut-off was arbitrary, but beyond normal travel distances by beetles. A site was visited for sampling during a single day; windy days and rainy days were avoided; and the average temperature during the days on which trapping was conducted was 22 °C (range 13–29).

### 2.2. Recent Forest Fire History

The data on fire history used in this study were obtained from the Swedish Civil Contingencies Agency, to which most local fire services report their firefighting activities. These data were supplemented with data from archives held at local fire authorities. Many fires lacked a spatial reference, and they were given coordinates after consulting the staff at the fire authorities. Although the total area of each fire is known, its geographic extent is not. Hence, we assumed a circular extent with the coordinates recorded as the centre.

Our regional data contained all fires greater than 100 m^2^, an arbitrary cut-off, for the years 1999–2010 in Östergötland county in south-east Sweden. In addition, we included eleven municipalities in neighbouring counties. The total study area was 21,877 km^2^, of which 14,394 km^2^ was forested (61.2%). The total area of the 1419 recorded fires in 1998–2010 was 20.54 km^2^, which means that 0.14% of the forested area had burned during this time period. The total area burned varied between 0.13 and 8.2 km^2^ per year, and the number of fires varied from 31 to 208 per year.

### 2.3. Setup of Beetle Sampling

To potentially increase the catching of pyrophilic beetles, smoke was generated by a fire in a 200 L metal drum and the insects were caught by regularly scanning a black nylon net set up around the fire area [29]. The net had a 1 mm mesh size and a total size of 20 m × 1.5 m = 30 m^2^. Birch logs were used as fuel for the fire (birch is a common local fuel that produces a relatively long-lasting fire), and smoke was created by putting humid forest litter on top of the burning birch logs. The fire was lit at 10:00 h and burned freely until 11:00 h, when the litter was added on top of the burning wood, generating smoke, and insect collection started. The fire was then attended to through the day by intermittently adding more wood as well as litter for smoke. In order to avoid any differences in litter moisture and quality between the sites, the forest litter was almost exclusively taken from the same site (a coniferous forest).

The insects considered in this study were beetles (Coleoptera), a group which contains many pyrophilic species. Insects flying into the square net setup were collected and then preserved in 95% alcohol. Because of the handling time of insects, fire management and weather observations, the active time of catching was standardised to 45 min per hour. At 21:00 h, the fire was extinguished, and the sampling was terminated. However, to ensure similar sampling efforts over sites, we decided to use six catch periods between 14:00 h and 21:00 h (mostly the 6 h between 15:00 h and 20:00 h). There were two reasons for this. First, we failed to collect data for some time periods due to rain or practical difficulties (especially affecting sampling between 11:00 and 13:00). Second, data from eight sites were incomplete because the handling of samples (sorting and identifying) turned out to be too time-consuming for the resources available. To make samples comparable, samples from 11:00 h to 15:00 h were omitted from all sites (the time delimitation was partly chosen because the number of Coleoptera individuals proved to be higher after 15:00 h) [29]. Hence, the trapping effort corresponded to 4.5 h of afternoon–evening patrolling of the net per site.

### 2.4. Species Classification

Beetles were classified as fire-favoured according to [32,33]. This includes species with adaptations to detecting fire [4], those specialised to “fire fungi” and those who are rarely seen in non-burnt sites.

As a control group, beetles that are not fire-dependent were classified by one of us (NJ), a field entomologist with extensive field experience with both beetles and with saproxylic species. As an additional control group, obligate saproxylic beetles (species that need dead or dying wood to complete their lifecycle) were classified according to [34].

N.b. that these classifications were not mutually exclusive; e.g., a pyrophilic species could be classified also as saproxylic. It is expected that the occurrence of most saproxylic species would be dependent on the amount of old-growth forest or deadwood, but not on fire history.

### 2.5. Statistical Analyses

#### Spatiotemporal Connectivity

To analyse the occurrence of pyrophilic beetles in relation to the forest fire history at different spatial and temporal scales, we used the ln-transformed odds of a random (1) beetle and (2) species being pyrophilic as response variables in two generalised linear models (with normal distribution), with spatiotemporal connectivity as an explanatory variable. To do this, we extend a classical spatial connectivity measure, e.g., [35], with a temporal aspect, and model spatiotemporal connectivity of the smoke attraction trap *i* (*ST_i_*) as the following:

(1) $${ST}_{i}=\sum_{j=1}^{n}e^{-d_{ij}\times \alpha_{s}}{FA}_{j}\times e^{-T_{j}\times \alpha_{t}}$$

where *d_ij_* is the distance in kilometres between the attraction trap *i* and surrounding forest fires *j*, *FAj* is the log-transformed total burned area (m^2^) in fire *j*, *T_j_* is the time since fire and *n* is the total number of fires. We only included fires within 20 km from any trap, as this was the maximum distance for which we had data on *j* fires for all traps. The parameters *α_s_* and *α_t_* set the spatial and temporal scaling and were optimised (between 0 and 4) based on the deviance profile, e.g., [36]; i.e., we used the values that provided the best model fit. Corresponding analyses were also conducted for the two control groups: non-pyrophilic and obligate saproxylic beetles. All calculations were done in R.

## 3. Results

### 3.1. The Catch

In the group of 2220 Coleoptera involved, 166 species and 3 higher level taxa were identified. The most prominent families were Staphylinidae (60 species), Ptiliidae (20) and Latrididae (11). The most abundant beetles were *Atheta harwoodi* (511 individuals), *Acrotrichis insularis* (500) and *Atomaria lewisi* (285).

Thirteen species were classified as being pyrophilic (Table 1). An additional 22 taxa were considered non-fire-favoured (Table 1). Finally, 34 species were classified as obligate saproxylic (Table 2), i.e., forest species relying on deadwood. There were also almost 100 species that did not belong to these three classifications, but that were part of the calculation of the ln(odds).

### 3.2. Fire History

**Figure 2:**
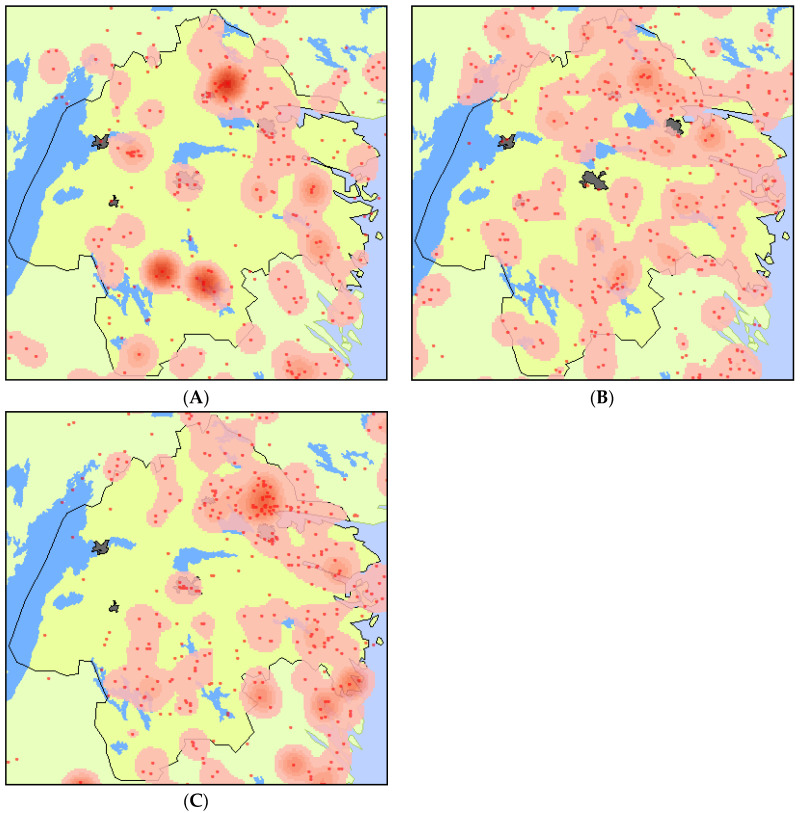
The forest fire history in Östergötland county divided into three time periods: 1–4 yrs prior to sampling (2007–2010) (**A**); 5–8 yrs (2003–2006) (**B**); and 9–12 yrs (1999–2002) (**C**). Each dot represents a fire. Darker areas show higher fire frequency, yellow indicate low fire frequency. Dark polygons indicate cities (only shown against the yellow background).

The density of fires 1–4, 5–8 and 9–12 years prior to the sampling showed that the forest fires were clustered to the east of Östergötland county, but with slightly different patterns for the three periods (Figure 2).

### 3.3. Spatiotemporal Connectivity

**Figure 3:**
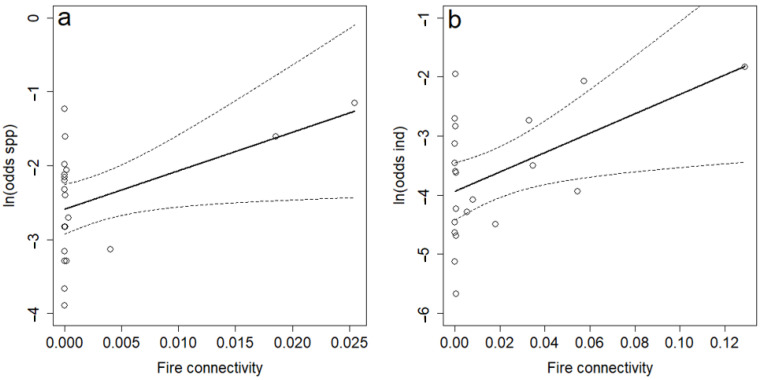
The relationship between spatiotemporal connectivity to fires and the ln(odds) of finding a pyrophilic beetle species (**a**) and individual (**b**) among the beetles in the smoke attraction traps. Circles represent trap sites. Regression line (solid) with confidence band (dotes lines) are indicated.

**Figure 4:**
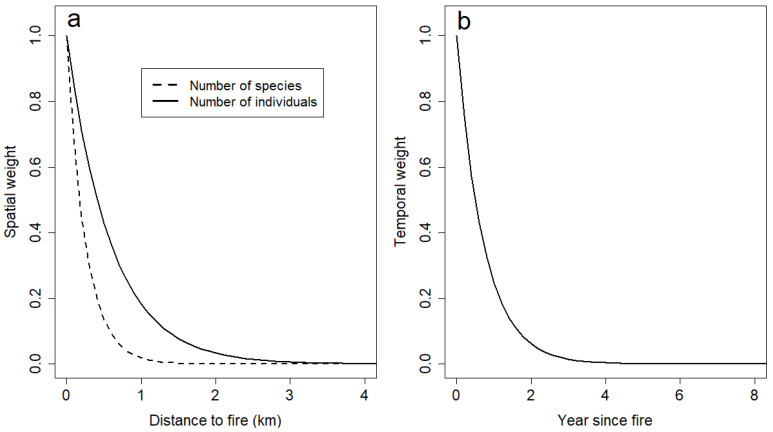
Weight of the contribution of fire *j* to the spatiotemporal fire connectivity of smoke attraction trap *i* (see Equation (1)). The spatial scaling parameter (*α_s_*) was 1.7 for individuals and 4.0 for species (**a**), while the temporal scaling parameter (*α_t_*) was 1.4 for both response variables (**b**).

Both the odds of finding a pyrophilic beetle individual and the odds of finding a pyrophilic species among the beetles in the smoke attraction traps increased significantly with increasing spatiotemporal connectivity to fires (Table 3 and Figure 3). The spatiotemporal scale that best explained the two response variables suggested a small spatial scale, with low weight given to fires beyond 2 km from the smoke attraction traps (Figure 4a), and a temporal scale, with low weight given to fires that occurred more than 2–3 years prior (Figure 4b). Non-pyrophilic beetles and saproxylic beetles did not have significant relationship with spatiotemporal connectivity, neither for species (p_min_ = 0.21) nor for individuals (p_min_ = 0.06).

## 4. Discussion

### 4.1. Spatial and Temporal Scales

The occurrence of pyrophilic beetles was associated with recent forest fires mainly in the smaller spatial scales, up to 2 km. Such scales seem smaller compared with those of a previous study on saproxylic beetles on oak [37], and more in line with distances recorded for butterflies in grasslands and forests [38], butterflies in wetlands and forests [39] or bees in grasslands [40]. As expected from their habitat and evolutionary history, some pyrophilic species can disperse relatively long distances. The authors of [18] calculated the average distance pyrophilous insects needed to travel for successive generations to breed in recent burns to be 30–60 km (based on recent fire history in Quebec). Few estimates are available regarding the dispersal capabilities of pyrophilous insects in the literature. Long dispersal distances have been attributed to the enigmatic *Melanophila acuminata*, but they remain contested [41,42]. One study [43] estimated that the pyrophilous beetle *Monochamus scutellatus* could travel over 10 km in its adult life, i.e., less than the 30–60 km needed according to [18]. If a beetle flies straight and at a steady speed of, say, 1 ms^−1^, it would travel 5 km in less than two hours. Thus, a spatial scale of up to several km is a reasonable starting point as an estimate for pyrophilous beetle assemblage.

One caveat of our study is the limited amount of smoke generated, and the fact that this was over a short time period (up to 12 h). If smoke attracts over longer distances, a longer smoking period is likely to have attracted more specimens, both due to the smoke plume affecting a larger area and by giving more time for specimens to fly in from longer distances [44,45]. Hence, more smoke for a longer period would have attracted more specimens and might have better reflected a natural forest, and possibly would have resulted in different estimates of spatial scales than when using a modest amount of attractant.

Fire events and the area burned in boreal forests tend to be highly aggregated in some years and nearly absent in others [46,47]. This suggests that the quality of the unburned matrix may be important in the population dynamics of fire-favoured insects. The detection of several beetle species exhibiting pyrophilous behaviour in recently dead trees in unburned forests supports this hypothesis [48]. Hence, even if burnt forest is the preferred or optimal habitat, the moderate response of pyrophilic species in the current study might be due to the importance of unburnt forest when this can function as an alternative habitat [49,50].

The time scale identified in this study seems somewhat shorter than that reported elsewhere for pyrophilous insects of forests in northern Europe. In a Polish study, pyrophilic species decreased during the 4–5 years after a fire [30]. In a Finnish study, Ground beetles sampled seven years after experimental fires [51] consisted of few species typical of newly burnt sites [52]. Furthermore, the abundance of pyrophilous flat bugs peaked after a few years [53], as did that of beetles considered both pyrophilous and saproxylic [54,55]. The post-fire dynamics of a pyrophilous species is likely subject to the abundance of key resources in the burnt patch as well as in nearby patches, as in a typical meta-population system [56].

### 4.2. Pyrophilic Species

There was a clear positive association between recent forest fire history and pyrophilic beetles. This was expected as burned forests maintain pyrophilic species for some years after a fire [14,15,16,17]. Several pyrophilous species are found at very high densities in recent burns but are uncommon or rare in unburned forests. However, the association between abundance of pyrophilic beetles as well as older forest fires (maximum 12 years) was weak or non-existent (unpublished data). This fits well with previous reports about the short-term effect on pyrophilic insects of a single fire, e.g., [51,53,54,55,57,58], and the notion that pyrophilic insects are particularly dependent on newly dead or dying trees. Some beetles have longer lifecycles, and there is also a possibility of using the burned habitat during more than one lifecycle, e.g., [23].

Furthermore, some pyrophilic beetles are dependent on pyrophilic fungi [7] that might peak in abundance with a short time-delay after the fire [59,60,61]. Together, this might answer the question of why pyrophilic beetles were associated with recent forest fires, but not with older fires. This has clear implications for the frequency of conservation burns. Although a fire might have long-lasting effects on beetle assemblages and biodiversity (e.g., [58]), the effect on pyrophilic species is short-term.

The richness of pyrophilic insects depends on fire history in the landscape [26], and it is worth pointing out that Östergötland might be low in the presence of pyrophilics compared with, e.g., northern Sweden [62]. Or, put another way, a study like ours conducted within an area with much more fires might have found even clearer results.

A surprising find was that two of the three most abundant species attracted by our sampling—*Atomaria lewisi* and *Acrotrichis insularis*—are considered detritivores (compost species) and happen to be non-native [15,63]. *Atomaria lewisi* has previously been recorded in abundance in burnt forest [15]. Given that recently burnt forest soil in Scandinavia is often blackened and covered with an abundance of shed needles, and there would be plenty of food for mycelium-feeders like these two species.

It is possibly surprising that the groups of obligate saproxylic species evaluated showed no association with fire; one of the defining features of a fire is the dead and dying wood created. Presumably, the amounts of deadwood created by fires in today’s forests are insignificant (that of a few fires, most of which are small) compared to what can be encountered at the landscape scale [64].

### 4.3. Sampling Efficacy

A methodological issue to consider is to what extent our trapping effort was effective, given that the attraction of beetles to the smoke is unclear [29], and the size and shape of the smoke plume are unknown. Only 2.7% of the specimens caught in the present study were of pyrophilic species. However, this low proportion was in line with that of pyrophilic species found at two conservation burns within our study area (1.8 and 2.1%; unpublished data combined from pitfall and window traps). Corresponding values for the number of pyrophilic species were 2.2 and 1.0% (unpublished data), while the current study considered 7.7% of the species pyrophilic. In northern Sweden, an area where forest fires have been more prevalent, refs. [10,58] both recorded 4% of species and 3% of specimens as pyrophilic. In an extensive study conducted in Finland, about 1% of the numerous species recorded were considered pyrophilous, and about 1% of the specimens [65]. So, on balance, considering that our study area might be poor in pyrophilic species [26,59], our systematic sampling in unburnt stands nevertheless trapped pyrophilic species well in line with what has been caught with flight intercept traps in burnt stands [58,62,63].

There are other limitations to the methods used that add noise to the data. For logistic reasons, sampling was spread out over time, meaning that slightly different assemblages of beetle species were flying from moment to moment. Also, weather is a crucial factor, causing smoke to attract in areas of different shapes and sizes. Also, it is worth noting that the amount of smoke generated was miniscule compared with that generated during a fire. Hence, it is possible that the smoke generated added little to our sampling [29].

### 4.4. Conclusions

Most beetle species considered pyrophilic were positively associated with recent forest fires, and with previous fires in the smaller spatial scales. The outcome of conservation burns, which are increasingly used in efforts by both private forest owners and the public sector in the boreal environment, can be increased by placing fires within a few km and years of previous fires. These narrow spatiotemporal scales suggest that spreading fires over the full landscape might not be a fruitful strategy, and that a more successful option is identifying subregions where conservation burns are likely to generate the greatest benefit for pyrophilic species based on recent fire history.

## Figures and Tables

**Table 1:** Groups of Coleoptera identified at 21 sites where smoke attraction trapping was conducted in Östergötland in 2011, and their abundance in the six catch periods between 14:00 and 21:00 h (i.e., 6 × 45 min × 21 sites = 94.5 h).

	Number of Individuals	Number of Species
Pyrophilic	60	13
Non-fire-favoured	227	22
Obligate saproxylic	67	34
Total	2220	169

**Table 2:** Species classified as pyrophilic, non-pyrophilic and obligate saproxylic.

	Family	Obligate Saproxylic Species	Occurrences (Max 21)	Number of Individuals
**Pyrophilic species**				
*Cartodere constricta*	Latridiidae		11	25
*Anthaxia quadripunctata*	Buprestidae	x	4	4
*Cortinicaria gibbose*	Latridiidae		3	12
*Corticaria ferruginea*	Latridiidae		3	8
*Corticarina fuscula*	Latridiidae		2	2
*Atomaria pusilla*	Cryptophagidae		1	16
*Phloeonomus pusillus*	Staphylinidae	x	1	2
*Melanophila acuminata*	Buprestidae	x	1	2
*Atomaria pulchra*	Cryptophagidae	x	1	1
*Littargus connexus*	Mycetophagidae	x	1	1
*Platystomos albinus*	Anthribidae	x	1	1
*Glischrochilus hortensis*	Nitidulidae		1	1
*Henoticus serratus*	Cryptophagidae		1	1
**Non-pyrophilic species**				
*Cyphon coarctatus*	Scirtidae		14	74
*Cyphon palustris*	Scirtidae		13	102
*indet Cantharidae*	Cantharidae		9	14
*indet Chrysomelidae*	Chrysomelidae		5	5
*Helophorus spp*	Helophoridae		4	6
*Brachypterus urticae*	Kateretidae		4	4
*Megasternum obscurum*	Hydrophilidae		2	4
*Cercyon sternalis*	Hydrophilidae		2	3
*Microcara testacea*	Scirtidae		2	3
*Cyphon punctipennis*	Scirtidae		2	2
*Athous niger/hirtus*	Elateridae		2	2
*Meligethes spp*	Nitidulidae		2	2
*Cryptopleurum subtile*	Hydrophilidae		1	3
*indet Coccinellidae*	Coccinellidae		1	2
*Cis lineatocribratus*	Ciidae	x	1	1
*Octotemnus glabriculus*	Ciidae	x	1	1
*Bradycellus verbasci*	Carabidae		1	1
*Cercyon pygmaeus*	Hydrophilidae		1	1
*Carcinops pumilio*	Histeridae		1	1
*Cyphon padi*	Scirtidae		1	1
*Athous haemorrhoidalis*	Elateridae		1	1
*Lagria hirta*	Tenebrionidae		1	1
**Other species:**				
*Anaspis rufilabris*	Scraptiidae	x	8	8
*Pteryx suturalis*	Ptiliidae	x	3	5
*Anaspis thoracica*	Scraptiidae	x	3	3
*Orthoperus nigrescens*	Corylophidae	x	2	5
*Dasytes plumbeus*	Dasytidae	x	2	4
*Pyropterus nigroruber*	Lycidae	x	2	2
*Alosterna tabacicolor*	Cerambycidae	x	2	2
*Pityogenes chalcographus*	Curculionidae	x	2	2
*Dryocoetes autographus*	Curculionidae	x	2	2
*Rhizophagus nitidulus*	Monotomidae	x	1	3
*Anoplodera sanguinolenta*	Cerambycidae	x	1	2
*Crypturgus subcribrosus*	Curculionidae	x	1	2
*Ptinella denticollis*	Ptiliidae	x	1	1
*Anisotoma orbicularis*	Leiodidae	x	1	1
*Anobium rufipes*	Ptinidae	x	1	1
*Epuraea pallescens*	Nitidulidae	x	1	1
*Epuraea angustula*	Nitidulidae	x	1	1
*Cryptolestes abietis*	Laemophloeidae	x	1	1
*Triplax russica*	Erotylidae	x	1	1
*Anaspis flava*	Scraptiidae	x	1	1
*Anoplodera maculicornis*	Cerambycidae	x	1	1
*Anoplodera rubra*	Cerambycidae	x	1	1
*Leptura quadrifasciata*	Cerambycidae	x	1	1
*Leptura melanura*	Cerambycidae	x	1	1
*Scolytus intricatus*	Curculionidae	x	1	1
*Dryocoetes hectographus*	Curculionidae	x	1	1

**Table 3:** The standardised parameter estimates (with SE) and *p*-values for the models of the ln(odds) of finding a pyrophilic beetle species or individual among the beetles in the smoke attraction traps; similar estimates for the two control groups: obligate saproxylic beetles and non-fire-favoured species.

	Species		Individuals	
	Estimate	*p*-Value	Estimate	*p*-Value
**Pyrophilic species**				
Intercept	−2.47 (0.15)	<0.001	−3.67 (0.20)	<0.001
Spatiotemporal connectivity	0.35 (0.16)	0.037	0.52 (0.21)	0.023
**Saproxylic species**				
Intercept	−1.87 (0.21)	<0.001	−3.27 (0.21)	<0.001
Spatiotemporal connectivity	0.17 (0.15)	0.27	0.41 (0.22)	0.07
**Non-pyrophilic species**				
Intercept	−1.42 (0.16)	<0.001	−2.03 (0.36)	<0.001
Spatiotemporal connectivity	0.21 (0.16)	0.21	0.74 (0.36)	0.06

## Data Availability

Data are available at ZENODO https://doi.org/10.5281/zenodo.13896087.
